# A neuroscientific hypothesis on the physical nature of consciousness

**DOI:** 10.3389/fnhum.2026.1758344

**Published:** 2026-03-12

**Authors:** Chirapat Ukachoke

**Affiliations:** Neurology Center, Phyathai 3 Hospital, Bangkok, Thailand

**Keywords:** consciousness, explanatory gap, GWT, hard problem, information, NCC, neural correlates of consciousness, qualia

## Abstract

This study addresses a central question in neuroscience: What is the physical nature of consciousness? Taking a neuroscientific approach, the study first establishes working definitions for its principal terms—qualia, consciousness, and information—to specify clearly the entities under investigation. It then analyzes the essential features of these defined terms. Information, in particular, is examined in detail across several aspects, including its carrier, nature, effects, interpretations, and meanings. The study next investigates the essential properties of consciousness and identifies potential entities that could underlie it. Candidate entities are drawn from two groups: physically established non-material entities in the brain, such as electrical fields, magnetic fields, electromagnetic waves, and neural information, and physically unestablished non-material entities proposed in various theoretical models. Each candidate is assessed for whether it can satisfy the required properties of consciousness. The analysis finds that the entity that most parsimoniously meets these criteria, without invoking new forces or physical laws, is neural information. Accordingly, the study proposes the hypothesis that consciousness is a form of neural information, specifically information encoded in the spatiotemporal patterns of electrochemical signaling within certain neural circuits. It presents empirically verifiable predictions derived from this hypothesis, making the hypothesis falsifiable. Further, it identifies a neural mechanism by which some information can manifest phenomenally as consciousness, enabling the occurrence of consciousness in the brain, and another mechanism underlying why this manifestation occurs only from the first-person perspective of some neural circuits. The study then compares its hypothesis and proposed mechanisms with existing theories of consciousness, clarifying how it differs in focus, explanatory scope, and thesis. Broader implications for neuroscience, clinical research, and the possibility of artificial consciousness are discussed, along with limitations of the present framework. Overall, because its evidence and arguments lie entirely within established neuroscience, with no novel entities, forces, or physical laws posited, this study advances a parsimonious and neuroscientifically grounded hypothesis on the physical nature of consciousness.

## 1 Introduction

Consciousness presents one of the deepest puzzles in neuroscience. Although we observe and experience it every day, we still do not know what it is physically, or how and why it occurs in the brain. Understanding these issues is of fundamental importance, both theoretically and practically. Theoretically, identifying its physical nature—whether it is a known physical entity or a novel one—will advance our understanding of nature and refine our neuroscientific and fundamental physical theories. Practically, such knowledge could guide the development of instruments that objectively detect, measure, and monitor consciousness, especially in unresponsive individuals, and help identify interventions that more effectively restore consciousness in those with impairments. Neuroscience, whose mission is to uncover the nature of all observable phenomena in the nervous system, therefore bears the responsibility of addressing and ultimately solving this profound question.

Historically, the problem of consciousness was almost exclusively within the domain of philosophy (Chalmers, 1995; Velmans, 2009b; Van Gulick, 2017). Since the 1990s, however, it has become a legitimate target of empirical science. With the development of robust experimental paradigms (e.g., masking, priming, and suppression) and advanced investigative methods (e.g., functional magnetic resonance imaging, magnetoencephalography, and intracortical recording), scientific research on consciousness has proliferated (Searle, 1998; Boly et al., 2013; Lamme, 2018; Seth, 2018; Del Pin et al., 2021; Signorelli et al., 2021; Storm et al., 2024). Numerous neuroscientific theories and conceptual frameworks have been proposed. Representative examples include the Global Workspace Theory (Baars, 1988, 2005; Baars et al., 2013, 2021), the Neurobiological Theory of Consciousness (Crick and Koch, 1990), the Recurrent Processing Theory (Lamme and Roelfsema, 2000; Lamme, 2006, 2010, 2018), the Integrated Information Theory (Tononi, 2004, 2012; Oizumi et al., 2014; Tononi et al., 2016), and the Predictive Processing Theory (Clark, 2013; Hohwy and Seth, 2020). Although these frameworks differ in their term definitions, investigation targets, and study approaches (Del Pin et al., 2021; Doerig et al., 2021; Sattin et al., 2021; Signorelli et al., 2021; Seth and Bayne, 2022; Storm et al., 2024; Cogitate Consortium et al., 2025), they have substantially advanced our overall understanding of consciousness (Lamme, 2018; Seth, 2018; Sattin et al., 2021).

Despite this progress, most theories of consciousness still focus on identifying the neural correlates of consciousness (NCC)—the minimal neuronal mechanisms jointly sufficient for producing a specific conscious experience (Crick and Koch, 1990, 1998; Chalmers, 2000; Block, 2005; Tononi and Koch, 2008; Koch et al., 2016; Owen and Guta, 2019). These theories primarily aim to determine which neural circuits and processes give rise to consciousness rather than what consciousness itself physically is. It remains unknown whether consciousness is a new kind of physical entity that emerges from the NCC or a known physical entity inherent within the NCC but not yet recognized. An analogy is a movie displayed on a television screen: The movie consists of dynamic images and sounds that we perceive, and although it is produced by the screen, it is not the screen itself. Likewise, although consciousness is produced by the NCC, it is not identical to them. What, then, is its physical nature, and how does it occur in the brain—issues often framed as the hard problem of consciousness (Chalmers, 1995; Libet, 1996; Velmans, 2009b; Weisberg, 2012; Van Gulick, 2017) and the explanatory gap (Levine, 1983; Chalmers, 2006; Block, 2009; Feinberg and Mallatt, 2020)? Notably, compared with progress in identifying the NCC, advances in comprehending these two problems have been relatively limited (Weisberg, 2012; Van Gulick, 2017; Wagner-Altendorf, 2024).

Therefore, this study seeks to address these fundamental questions: what consciousness physically is and how it occurs in the brain. It will mainly employ a neuroscientific approach, emphasizing evidence and concepts grounded in neuroscience, while remaining informed by and in dialogue with relevant philosophical and psychological analysis. Further specifying the exact characteristics of the NCC is not the objective of this study and will not be attempted.

## 2 Definitions

“Qualia,” “consciousness,” and “information” are the principal terms used in this study. However, their meanings vary across the literature, which can lead to confusion (Rosenthal, 2009; Velmans, 2009a; Sattin et al., 2021). Since there are no standard definitions for these terms, this article establishes working definitions for them. The study employs definitions that refer to phenomena that are easily and commonly recognized in everyday life, not to theoretical constructs that are difficult to understand. These working definitions are as follows:

### 2.1 Qualia (singular: quale)

Qualia are phenomena that manifest what they are like, or manifest phenomenally, in the mind (based on Flanagan, 1992, pp. 61–85; Block, 1995; Koch, 2004; Kanai and Tsuchiya, 2012; Ukachoke, 2024, pp. 54–61). As such, we can be aware of what they are like and, under normal conditions, can report their occurrences. For example, images, sounds, smells, emotions, or thoughts in our minds are qualia: They manifest what they are like in our minds, and we can be aware of what they are like and, normally, can report their occurrences. Current evidence indicates that qualia originate in some specific types of neural processing, such as visual (image), auditory (sound), or olfactory (smell) perception (Fitzpatrick and Mooney, 2018; Kandel and Shadlen, 2021). In contrast, qualia do not occur in other types of neural processing, such as the interoception of blood constituents, cerebellar coordination, or autonomic regulation. Nothing in these processes manifests what it is like. Thus, even though the brain continuously monitors them, we can neither be aware of what anything in them is like, nor can we report their occurrences (Velmans, 1991; Bargh and Morsella, 2008; Hassin, 2013; Dehaene, 2014, pp. 47–88). For instance, we are not aware of what it is like to sense a plasma sodium level at a certain value, to accurately coordinate countless muscle fibers in various body parts to walk securely, or to control cardiac contraction to maintain blood pressure, nor can we report that these processes occur. Accordingly, nothing in these processes is a quale.

Although we do not yet know what a quale physically is, we know that it normally represents something the brain is engaging with—whether external or internal—such as an object being seen, an air vibration being heard, or a neural processing product (e.g., a thought, memory, or emotion) being experienced (Ramachandran and Hirstein, 1997; Feinberg, 2012; Feinberg and Mallatt, 2016; Tye, 2017; Lycan, 2023).

### 2.2 Consciousness (adjective: conscious)

Consciousness is awareness of what something is like (based on Block, 1995; Chalmers, 1996, pp. 3–31; Zeman, 2001; Koch, 2004; Ukachoke, 2024, pp. 61–65, 106–114). For instance, to be conscious of an image, sound, smell, emotion, or thought is to be aware of what that image, sound, smell, emotion, or thought is like, respectively. Such awareness is typically accompanied by an experience (Chalmers, 1995). Thus, in total, consciousness is both awareness and an experience of what something is like. Since “something” in this case manifests what it is like, it is a quale. Accordingly, consciousness is awareness and an experience of what a quale is like. This distinction is based on evidence. We cannot be aware of or experience physical things or their activities—such as an object, air vibration, molecules contacting our tongue or nasal mucosa—directly. Instead, we can only be aware of and experience their representations in the forms of qualia, such as visual (image), auditory (sound), gustatory (taste), or olfactory (smell) qualia. Notably, in real life, we normally are aware of and experience several qualia at the same time. For example, right now, the reader is aware of and experiences the image and sound, and even smell, qualia of the surroundings, as well as the thought quale in the reader’s mind. Therefore, consciousness is typically awareness and experiences of what qualia are like (Chalmers, 1995).

Crucially, since consciousness itself manifests phenomenally—evidenced by the fact that we can be aware of and experience what our consciousness is like and can report its occurrence—it can be regarded as a type of quale (Nagel, 1974; Chalmers, 1995; Searle, 1998; Koch, 2004). However, it is a quale of a special kind: It represents the neural awareness and experiences of what qualia, including itself, are like (Peters, 2014). Since qualia are already forms of representation, consciousness is thus a distinctive form of representation—specifically, a higher-order representation (Rosenthal, 2006; Gennaro, 2018; Brown et al., 2019; Carruthers and Gennaro, 2023) that also involves self-representation (Kriegel, 2009; McClelland, 2018).

Consciousness by this kind of definition is usually referred to as phenomenal consciousness (Block, 1995; Chalmers, 1996, pp. 3–31; Zeman, 2001; Koch, 2004; Ukachoke, 2024, pp. 61–65, 106–114). However, in the literature, the term “consciousness” can refer to various mental aspects, states, or functions that, although related to, differ from phenomenal consciousness (Zeman, 2001; Rosenthal, 2009; Velmans, 2009a; De Sousa, 2013; Van Gulick, 2017). Importantly, consciousness in this definition does not refer to alertness—the mental responsiveness to stimuli. Also, it does not refer to the mental state when its content has entered the so-called global workspace and become available for processing by multiple cognitive systems (e.g., analysis, communication, and motor control). This kind of mental state is usually referred to as access consciousness (Block, 1995; Chalmers, 1996, pp. 228–229; Rosenthal, 2009; De Sousa, 2013; Van Gulick, 2017). Additionally, consciousness by this definition is not, and does not consist of, other cognitive functions such as perception, attention, decision-making, learning, or action selection. These cognitive functions can exist in various forms in other animal species (Roth and Dicke, 2005; Butler, 2008; Edelman and Seth, 2009; Boly et al., 2013), robots, and other artificial intelligence (AI) systems (Kotseruba and Tsotsos, 2020; Ilić and Gignac, 2024; Oyeniyi, 2024). Although these cognitive functions in humans pose difficult empirical questions—what the exact circuits and processes underlying them are—they do not present a conceptual challenge. Theoretically, certain algorithms implemented in physical systems can produce such functions (Lamme, 2018). Nowadays, we routinely construct such physical algorithms in non-human systems, such as electronic ones, creating various forms of AI with these cognitive capabilities. By contrast, consciousness as defined above—awareness and experiences of what qualia are like—is special: It manifests phenomenally and, based on existing evidence, likely does not occur in plants, simple animals, and robots (Dehaene et al., 2017; Feinberg and Mallatt, 2020; Mallatt and Feinberg, 2021). Currently, we do not understand, even in principle, how some physical processes or algorithms can produce it and cannot yet artificially create it (Korteling et al., 2021; Zhao et al., 2022). It poses one of the most fundamental problems in science and is the focus of this study.

Finally, because consciousness is a kind of quale, this study investigates the nature of both consciousness and qualia in general. At times, for conciseness, one will be discussed as a prototype.

### 2.3 Information

Information is central to understanding the nature of qualia and consciousness. It has several aspects that must be discussed in detail before the problem can be addressed effectively. The information we encounter in our everyday lives can be defined as something *non-material* ^1^ consisting of physically transferable and causally effective *content*^2^ (based on Bateson, 1972; Landauer, 1999; Floridi, 2005, 2009; Deutsch and Marletto, 2015; Dubrovsky, 2019; Krzanowski, 2020; Ukachoke, 2024, pp. 181–197).

For example, the information on this page is non-material, consisting of content (about definitions of terms) that is physically transferable (from the author to the page, then to the reader) and causally effective (causing some specific pattern of dots or pixels on the page and certain cognitive activities in the reader, e.g., comprehending, analyzing, and remembering it). Similarly, the information in a television broadcast is non-material, consisting of content (e.g., about a movie) that is physically transferable (from the television station to the broadcasted electromagnetic [EM] waves, to television sets, then to viewers) and causally effective (e.g., resulting in a specific pattern of the broadcasted EM waves, producing the movie’s images and sounds on the television screens, and generating audio-visual experiences of the movie in the viewers).

#### 2.3.1 Information’s carrier

Because information content is transferable between physical entities, information itself exists in a physical medium (Landauer, 1996, 1999; Floridi, 2005; Deutsch and Marletto, 2015; Dubrovsky, 2019; Krzanowski, 2020; Wang, 2022), referred to as the information’s carrier (Floridi, 2005; Dubrovsky, 2019; Krzanowski, 2020). For instance, the information on this page is carried by dots or pixels, while the information in a television broadcast is carried by EM waves.

#### 2.3.2 Information’s nature

When we examine the information present in the various things around us, we can conclude that information is the spatiotemporal pattern of its carrier—more precisely, the organization in space and time of all the carrier’s physical components (Bates, 2005, 2006; Meijer, 2013; Orpwood, 2017; Krzanowski, 2020). It is only through this pattern that a piece of information occurs, exists, changes, and disappears—simultaneously and correspondingly. For example, the information on this page is the spatial pattern (without a temporal component) of its dots or pixels; the information in a television broadcast is the spatiotemporal pattern of the electromagnetic waveforms; and the information in a voice announcement is the spatiotemporal pattern of its sound waves. In all these cases, the occurrence, existence, change, and disappearance of information are completely tied to the spatiotemporal pattern of its carrier.

To avoid possible conflation, it is important to note that information is not its physical carrier or its carrier’s physical components, which are material, but rather a non-material pattern instantiated by that carrier’s physical components. For instance, the information on this page is the spatial pattern of the page’s dots or pixels, but not the dots or pixels themselves.

#### 2.3.3 Information’s effects

Crucially, the effects of a piece of information—that is, the effects of a particular spatiotemporal pattern—are neither fixed nor absolute. They depend on the information receiver and on the context in which the information is transferred (Meijer, 2013; Krzanowski, 2020). For example, the effects of the dot-or-pixel pattern “意识” are not fixed or absolute. They vary across different receivers: people who understand Chinese, people unfamiliar with Chinese, nonhuman animals, optical devices, and so on. For Chinese-literate individuals and appropriate optical devices, the pattern results in meaningful effects, leading to noticeable outcomes—such as pronouncing the word aloud or generating synthesized speech of the word. For others, it is meaningless, producing trivial or negligible outputs. Even among Chinese speakers, the effects depend on the context of the information transfer. In different situations, they may take different actions, depending on what the pattern means to them, such as “consciousness,” “awareness,” or “awakening” (Cambridge Dictionary, 2025). This is discussed next.

#### 2.3.4 Information’s interpretations and meanings

In this article, the information receiver *interprets* a piece of information as M, and that information *means* M to the receiver, if the information causes the receiver to *incorporate M into the receiver’s subsequent activities* (based on Floridi, 2005; Bates, 2006; Meijer, 2013; Krzanowski, 2020). For example, if the information carried by “意识” leads a receiver to include “consciousness” into the receiver’s subsequent activities—such as reading the word aloud, remembering it, or writing it as “consciousness”—then the receiver must interpret the information as “consciousness,” and the information means “consciousness” to that receiver. In a different context, if it causes a receiver to integrate “awareness” into the receiver’s succeeding activities—such as reading it aloud, remembering it, or writing it as “awareness”—then the receiver must interpret it as “awareness,” and it means “awareness” to that receiver. Yet in another context, if this information causes a receiver to take it as nothing and disregard it, then the receiver must interpret it as nothing, and it means nothing to that receiver.

Like the effects of a piece of information, the interpretation and meaning of a piece of information are not fixed but depend on the information receiver and the context in which the information is transferred (Meijer, 2013; Krzanowski, 2020). For instance, the interpretation and meaning of the dot-or-pixel pattern “意识” are not fixed or absolute but depend on the context of the information transfer, as exemplified above.

Next, is a seminal point. Because the effects, interpretation, and meaning of a piece of information depend on the information itself, the information receiver, and the context of information transfer, a piece of information does not have inherent effects, interpretation, or meaning. Because, for a certain piece of information, these last two factors are variable, the effects, interpretation, and meaning of a piece of information are not fixed—they vary with these two determining factors. Therefore, although a physical carrier has an established pattern and even though the information carried by that physical carrier is that pattern, the information in that pattern does not have fixed or absolute effects, interpretation, or meaning (Bates, 2005, 2006; Meijer, 2013; Krzanowski, 2020; Wang, 2022).

Because the pattern of a physical entity, the nature of the receiver, and the context of information transfer can, theoretically, vary infinitely, it is in principle possible for information to mean anything to some receivers under suitable circumstances. Thus, theoretically, certain information—certain patterns of certain physical carriers—can mean a phenomenal quale or consciousness of something to some receivers in appropriate contexts and causes those receivers to incorporate something about that quale or consciousness into their subsequent activities, such as considering, comparing, and categorizing that quale’s or consciousness’s characteristics.

In the brain, patterns of neural signaling are also a form of information. This fact—combined with the observation that some information can mean a quale or consciousness of something to some receivers in certain circumstances—is crucial to understanding the physical nature of qualia and consciousness. This will be explored in the following sections.

## 3 Essential properties of qualia and consciousness

Because, in general, the nature of something can be determined from its properties, one way to discover the nature of qualia and consciousness is to examine their properties. However, since a range of their properties has been proposed by several authors, investigating all of them is not feasible. Therefore, this article examines only those it considers essential. They are as follows:

### 3.1 Materiality property

Because qualia and consciousness are macroscopic phenomena (observable at the brain level) that can appear, change, and disappear within milliseconds (Metzinger, 2003; Dehaene, 2014, pp. 115–160; Lamme, 2018) without consuming or giving off enormous amount of energy in the process and because no macroscopic material entities have this property, we can conclude that qualia and consciousness are non-material (see text Footnote 1). Thus, they can be some forms of electrical fields, magnetic fields, electromagnetic waves (EM waves) (McFadden, 2002, 2020, 2023; Pockett, 2012; Jones, 2019, 2024; MacIver, 2022; Sun, 2022; Ward and Guevara, 2022; Hunt et al., 2024), information (Tegmark, 2007; Orpwood, 2017; Dubrovsky, 2019; Ukachoke, 2024, pp. 87–100, 125–131), or other non-material phenomena.

### 3.2 Spatial existence property

Qualia and consciousness (as defined) are found only in the brain; they have never been found in other organs or tissues (e.g., blood, bone, skin) or things outside the brain. This is evidenced by the fact that these organs, tissues, or things outside the brain can be destroyed or changed without affecting one’s qualia or consciousness. On the other hand, when some part of the brain is affected or destroyed while everything else remains the same, one’s quale or consciousness is affected or destroyed (Ukachoke, 2024, pp. 22–24). Therefore, we can conclude that they are something existing within the brain.

### 3.3 Neural-processing association property

Current evidence shows that qualia and consciousness occur only when certain neural circuits engage in particular kinds of processing. They do not arise by themselves or from metabolic activity, genetic processes, or structural modification of circuits—even though these processes support neural function. If these non-neural processes remain constant but some particular neural processing event begins, changes, or ceases, the corresponding quale or conscious state appears, changes, or disappears (Searle, 2000; Edelman, 2003; De Sousa, 2013; Fitzpatrick and Mooney, 2018; Purves and Platt, 2018; Kandel and Shadlen, 2021).

Thus, qualia and consciousness are non-material phenomena generated by or closely associated with particular kinds of neural processing.

### 3.4 Neural processability property

This property is their cardinal property: Consciousness and qualia are neurally processable, yielding neural activities related to them. This is evidenced by the fact that, after a quale or consciousness appears, the brain can be aware of its occurrence, experience what it is like, and exhibit other activities specific to it, such as recognizing, remembering, and reporting its occurrence and characteristics. Because being aware of and experiencing a quale, as well as recognizing, remembering, and reporting the quale, requires neural processing of the quale, the quale is processable by neural circuits—that is, it is neurally processable. Otherwise, awareness, experiences, and other neural activities about it would not occur, which is not the case (based on Fitzpatrick and Mooney, 2018; Purves and Platt, 2018; Seth and Baars, 2005).

In summary, qualia and consciousness are *neurally processable, yielding neural activities related to them.*

### 3.5 Phenomenal property

As discussed in Sections 2.1 and 2.2, qualia and consciousness manifest phenomenally—they manifest what they are like in the mind—enabling us to be aware of, experience, report, and perform other cognitive activities regarding what they are like (Flanagan, 1992, pp. 61–85; Block, 1995; Koch, 2004; Kanai and Tsuchiya, 2012; Ukachoke, 2024, pp. 54–61). For instance, a flower visual quale manifests what its shape, color, and brightness are like in the mind—we can be aware of and experience what these phenomenal manifestations are like. It should be noted that this property poses a fundamental problem in science: How can something manifest phenomenally in physical systems? It will be discussed more in Section 6.1.

### 3.6 Differential appearance property

Qualia and consciousness exhibit differential appearances among the first and third persons: They manifest phenomenally in the first-person view but non-phenomenally in the third-person view (Van Gulick, 2017; Tyler, 2020). For example, a flower visual quale appears only in the person who looks at the flower and has the first-person view of it; it does not appear in the third-person view of other people who look at the brain of that first person. Like the phenomenal property, this property poses a fundamental problem to science: How does something manifest phenomenally in the first person but not in the third person? It will be discussed further in Section 6.2.

### 3.7 Representational property

As noted in Sections 2.1 and 2.2, excluding pathological cases (such as migraine auras, epileptic auras, and hallucinations), a quale, as well as consciousness, typically represents something the brain is dealing with (Ramachandran and Hirstein, 1997; Metzinger, 2003; Seth and Baars, 2005; Seth et al., 2006; Van Gulick, 2017), although that representation may sometimes be inaccurate and result in illusions. For instance, an image quale of a flower represents the flower being seen, and consciousness of the flower’s image quale represents the neural awareness and experience of the image quale of the flower. Thus, under normal conditions, qualia and consciousness serve representational functions.

### 3.8 Other properties

#### 3.8.1 Intrinsic, private, and subjective

Qualia and consciousness are intrinsic to the individual, private, and inherently subjective (Edelman, 2003; Seth and Baars, 2005; Seth et al., 2006; De Sousa, 2013; Feinberg and Mallatt, 2016; Tye, 2017; Van Gulick, 2017; Tyler, 2020). No one else can directly access another person’s qualia or consciousness.

#### 3.8.2 Ineffable or indescribable

Qualia have characteristics that cannot be exhaustively described based on other phenomena. For example, the phenomenal nature of color cannot be conveyed to someone congenitally blind through descriptions of sounds, smells, or touches (Tye, 2017; Van Gulick, 2017; Lycan, 2023).

#### 3.8.3 Complex, differentiated, and diverse in modality and content

Qualia and consciousness can be complex and highly differentiated and can vary widely in modality (e.g., visual, auditory, olfactory, emotional, and cognitive) and content (e.g., from an image of total darkness to an image of a dazzling city center) (Edelman, 2003; Seth and Baars, 2005; Seth et al., 2006; Seth, 2009; Tyler, 2020).

These properties will be discussed in more detail in Section 6.4. More properties can be found in the cited references.

Thus, we can have a general picture that qualia and consciousness are something non-material that exists in the brain and possesses other properties as outlined above. One way to identify what they specifically are is by finding the entity with similar properties (Chalmers, 1995, 1997; Ukachoke, 2024, pp. 87–98).

## 4 Two groups of non-material entities in the brain

Within the context of brain function, we can distinguish two mutually exclusive groups of non-material entities, one of which may underlie qualia and consciousness. The groups are as follows:

### 4.1 Group I: Physically established non-material entities

These include electric fields, magnetic fields, EM waves, and information. Since all entities in this group are physical, if qualia and consciousness are some forms of them, they will also be physical and thus governed and explicable by physical laws. Our tasks are to identify which entity underlies qualia and consciousness; no additional physical laws need to be posited to govern their interactions with the physical world.

### 4.2 Group II: Physically unestablished non-material entities

This group consists of entities or fundamental properties that are hypothesized and currently remain outside the present physical framework. These include:

1. Psychons (Eccles, 1992): hypothetical non-physical mental units proposed to interact with neuronal activity, influencing neural firing by injecting “mental intentions” into synapses.
2. Conscious Mental Fields (Libet, 1996): a proposed non-physical field that coexists with neural activity, thought to guide or modulate neural processes to generate consciousness.
3. Novel fundamental elements: entities or properties existing at a deeper level than presently known physical reality. They are posited by frameworks such as neutral monism (Silberstein, 2009; Silberstein and Stuckey, 2020; Stubenberg and Wishon, 2023), dual-aspect theory (Atmanspacher, 2014), and panpsychism (Brüntrup, 2016; Chalmers, 2016; Goff and Moran, 2021; Goff et al., 2022). These may be:

- Neutral entities that differentiate into physical and mental aspects.
- Intrinsic dual-aspect properties manifesting as either physical or phenomenal, depending on the perspective.
- Proto-conscious units inherent in all matter.

Since all these proposed entities are theoretical and outside the current physical framework, if any of them underlie qualia and consciousness, new hypotheses would likely be required to govern how qualia and consciousness interact with the physical world.

## 5 The physical nature of qualia and consciousness

As noted at the end of Section 3, one way to identify the physical nature of qualia and consciousness is to find an entity with the essential properties of qualia and consciousness. This can be achieved more readily by studying the functioning of neural circuits involved in the generation and manifestation of qualia and consciousness and finding an entity that possesses some of the essential properties, including the cardinal property, first. Whether that entity also has all other essential properties will be verified later.

**Figure 1:**
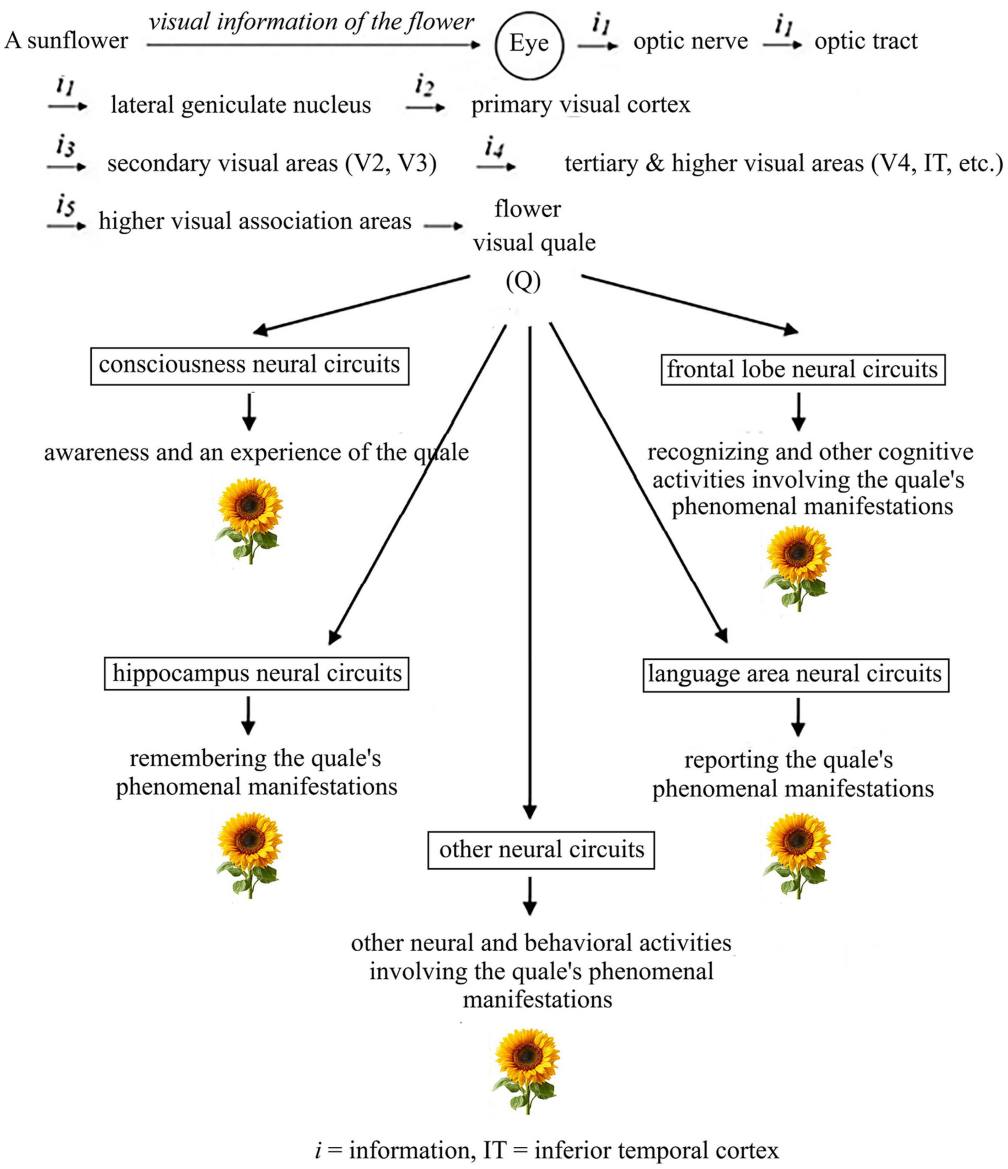
A quale emerges in visual perception through stages of neural information processing, followed by various neural activities about it.

As a representation of qualia and consciousness in general, this study investigates a typical case of a visual quale occurring when one sees a flower. Consider Figure 1. When one sees a sunflower, its visual information, carried by the reflected light, enters the brain through the eyes. The first stage of processing occurs in the retinas, followed by further processing in successive visual centers: the lateral geniculate nucleus, areas V1, V2, V3, V4, and so on (Fitzpatrick and Mooney, 2018; Albright and Freiwald, 2021; Gilbert and Das, 2021; Meister and Tessier-Lavigne, 2021). Eventually, the flower visual quale (Q) emerges from the neural processing, and the person becomes aware of its occurrence and experiences its phenomenal manifestations—shape, color, brightness, etc. Other neural activities involving the quale and its phenomenal manifestations, such as recognizing, remembering, and reporting its shape, color, and brightness, can also follow.

The question is: What is the physical nature of the flower visual quale (Q)?

The answer can be obtained by analyzing the neural processing associated with it at this point:

1. Since Q occurs when a certain neural circuit group is processing, it is something created by or closely associated with that neural circuit group’s processing. This is Property 3.3, previously described.
2. Since after Q appears, various neural activities involving it occurs, such as awareness of its occurrence and experiences of its phenomenal manifestations (e.g., of its phenomenal shape, color, and brightness) as well as other downstream activities about it, such as recognizing, remembering, and reporting its phenomenal manifestations, Q must be something that neural circuits can detect, process, and generate outputs with something about it incorporated. That is, Q must be neurally processable, yielding neural activities related to it. This is Property 3.4, previously outlined.

Therefore, we can conclude that, minimally, Q is something with the above two properties.

### 5.1 Neural information

When the entities in Groups I and II are examined, it can be found that the only entity that naturally possesses the above two properties without having to formulate additional physical laws to support its possession is neural information.

This is because the nervous system has both apparatus and mechanisms to generate, detect, receive, and process neural information for its functions. Neural information is naturally generated in the nervous system all the time in various forms. One form is the patterns of action potentials in neural electrochemical signaling. This information is transmitted principally along the axons to other neurons in the circuit. The receiving neurons *read* ^3^ the information—the patterns of the action potentials—then process it and transmit their results to specific neurons in the circuit for further processing. Because of the evolved architecture of the circuit and of the distinctive way the information is processed when passing through the circuit, the circuit can produce appropriate functional outputs related to the original information (Augustine, 2018; Abbott et al., 2021; Amaral, 2021; Javitch and Sulzer, 2021; Koester and Siegelbaum, 2021; Shadlen and Kandel, 2021).

Therefore, if the flower visual quale Q is in this form of neural information—the patterns of action potentials in neural electrochemical signaling—it can naturally be generated by neural circuits and be processable, giving rise to meaningful outputs specific to it, such as recognizing, remembering, and reporting its occurrences and characteristics.

### 5.2 The other entities in Group I

For the other entities in Group I—electrical fields, magnetic fields, and EM waves—although they possess Properties 3.1 to 3.3 (being non-material, existing in the brain, and being generated by or closely associated with neural processing), none of them satisfy Property 3.4: being neurally processable and yielding neural activities related to them. This is because the nervous system has neither the apparatus nor the mechanisms to detect, receive, and process these entities in a way that generates functional outputs related to them, as demonstrated below.

- Absence of specialized receptors or transduction mechanisms.

Except for the photopigments in retinal rods and cones, no neurons or other cells in the nervous system possess comparable molecules or organelles capable of properly reacting with EM-wave, electrical-field, or magnetic-field signals to extract specific effects from them at some part of the neurons. These signals affect the whole neurons diffusely and nonspecifically, just as they do other cell types in the body.

- Lack of machinery to convert nonspecific effects into meaningful output signals.

Neurons lack cellular machinery and processes capable of processing and transforming such diffuse, nonspecific influences into discrete, meaningful output signals specific to the influences and of the same category (e.g., generating new functional EM-wave signals in response to countless input EM-wave signals, converging on them from surrounding neurons in all directions).

- Inability to emit or transmit these signals directionally.

Neurons do not have cellular machinery to emit resulting EM-wave, electrical-field, or magnetic-field signals directionally to target neurons, especially when those neurons are far apart (e.g., thalamus to primary sensory cortex, sensory cortices to frontal cortex, or motor cortex to spinal cord ventral horn). Any such signals radiate outward uncontrollably in all directions at light speed.

- Lack of circuit-level coordination mechanisms.

Neural circuits lack mechanisms to coordinate EM-wave, electrical-field, or magnetic-field signals emitted from their distributed neurons so as to generate coherent, circuit-level functional effects (based on Abbott et al., 2021; Amaral, 2021; Gardner and Gardner, 2021; Shadlen and Kandel, 2021)

From these observations, this study concludes that neural EM-wave, electrical-field, and magnetic-field signals cannot be considered neurally processable in the sense specified in Section 3.4. This is because they are not detected, received, and processed in a controlled manner, because they cannot be integrated to produce outputs related to them, and because their results cannot be specifically transmitted to target neurons or circuits.

However, critically, the above conclusion does not mean that neurally generated EM waves, electrical fields, or magnetic fields do not have functional influence on neural processing at all. Some empirical evidence suggests that neurally generated EM waves may play modulatory roles in long-term neural plasticity and circuit reconfiguration (Wang et al., 2019). Such effects, however, do not evidence that these signals themselves are processed and yield specific outcomes related to them as neural electrochemical signals are and do. Likewise, although electrical and magnetic field activity affect neurons and neural circuits and can be routinely recorded as local field potentials (Kajikawa and Schroeder, 2011; Einevoll et al., 2013; Herreras, 2016), electroencephalography (Mukamel and Fried, 2012; da Lopes Silva, 2013; Tatum, 2020), and magnetoencephalography (Proudfoot et al., 2014; Gross, 2019; Ebersole and Funke, 2020), and although these fields can, among other things, modulate action-potential transmission through ephaptic coupling (Anastassiou et al., 2011; Han et al., 2018; Montgomery, 2023) and could guide neocortical network activity (Fröhlich and McCormick, 2020), there is no evidence that their signals themselves are processed in the way electrochemical signals are, nor that they produce functional outputs specific to them as electrochemical signals do (Koester and Siegelbaum, 2021; Javitch and Sulzer, 2021; Shadlen and Kandel, 2021).

Additionally, it is important to note that neural electromagnetic, electric, and magnetic fields likely contain information about neural processing, such as firing rates, spike timing, and the specific neural populations activated, because neural processing consists of electromagnetic activity. However, due to the absence of neural apparatus and mechanisms capable of selectively extracting and processing specific information from these fields, the information they contain is not neurally processable in the sense required for generating downstream neural activities specific to particular qualia or conscious experiences, such as experiencing, recognizing, or remembering a flower visual quale. Therefore, although these fields may reflect information correlated with qualia or consciousness, there is currently no neuroscientific basis for considering them as the entities that underlie qualia or consciousness.

Regarding the unified appearance of qualia and consciousness, some accounts have suggested that electromagnetic field theories have an advantage in explaining phenomenal unity by appealing to the intrinsic spatial continuity of fields (see McFadden, 2002, 2020, 2023; Pockett, 2012; Jones, 2019, 2024). However, unity of consciousness does not necessarily require spatial continuity of a single physical medium. Unity can also arise through other means, such as coordinated informational relations among discrete components. For example, a linguistic expression (like “unified consciousness”) conveys a single, unified meaning to listeners, even though its physical realization consists of discrete and discontinuous elements (e.g., letters or sounds). Likewise, in the present framework, phenomenal unity is explained by the coordinated and integrated processing of neural signaling information across highly interconnected neural circuits. The brain’s dense connectivity and recurrent interactions allow large-scale neural signaling patterns to form high-dimensional informational states, capable of representing percepts as either fragmented or unified (see Section 6.4.3). Importantly, such informational unity is brain-state dependent and consistent with empirical observations of both unified and disrupted conscious experience, whereas electromagnetic fields remain spatially continuous across both conscious and disordered conscious states. Moreover, while classical EM fields are traditionally viewed as continuous, quantum field theory reveals a discrete underlying structure of photons, suggesting that even the apparent “field unity” may emerge from discontinuous quantum particles and their activity.

In conclusion, although EM-wave, electrical-field, or magnetic-field very probably contain information about neural processing and can modulate some neural processing, there is no evidence that their signals themselves are processed in any neural processing in such a way that output activities related to them are generated and sent to other neurons or neural circuits for further processing. Thus, if the flower quale Q or any quale is grounded in one of these entities, it would not be processable and would not yield subsequent activities specific to it. Neural activities associated with the quale—such as recognizing, remembering, and reporting its occurrences and characteristics—would not occur, which is contrary to fact. Therefore, this study concludes that these entities cannot underlie Q and that they are not required for qualia and consciousness to be unified, either.

### 5.3 Entities in Group II

Next, consider entities in Group II, or physically unestablished non-material entities. These include psychons (Eccles, 1992), conscious mental fields (Libet, 1996), and fundamental entities posited by neutral monism (Silberstein, 2009; Silberstein and Stuckey, 2020; Stubenberg and Wishon, 2023), dual aspect theory (Atmanspacher, 2014), and panpsychism (Brüntrup, 2016; Chalmers, 2016; Goff and Moran, 2021; Goff et al., 2022). While these entities could be postulated to satisfy Properties 3.2 and 3.3—existing in the brain and being closely associated with neural processing—there is no evidence that any of them can satisfy Property 3.4, namely, being neurally processable and resulting in neural activities related to them, without invoking additional physical laws. The rationale is as follows:

Like entities in Group I except information, there is currently no neuroscientific evidence showing that the nervous system has apparatus or mechanisms capable of systematically detecting, receiving, and processing something other than electrochemical signals to produce functional outputs related to them. Thus, if Q arose from some entity in Group II, it would not be systematically detectable, receivable, or processable by the neural system, and no outputs specific to it would be generated, which is not the case.

Thus, in light of current empirical evidence and present neuroscientific principles and based on the parsimony principle, this study deduces that these hypothetical entities, even if that they exist, do not underlie Q.

### 5.4 The preliminary hypothesis

Based on the preceding analysis and following the principle of parsimony, it is reasonable to infer at this stage that neural information underlies Q—the flower visual quale. Because this example is an ordinary instance of perception, the inference can be generalized to cover all forms of qualia and consciousness. Thus, this study preliminarily concludes that neural information is the entity that underlies qualia and consciousness and that every quale and every consciousness is a form of neural information. This conclusion is similar, though not identical in its exact formulation, to notions presented by several authors before (see Tegmark, 2007; Orpwood, 2017; Dubrovsky, 2019; Zhuravlev, 2023; Ukachoke, 2024, pp. 87–100, 125–131). As discussed in Section 5.1, the form of this information is specifically the patterns of action potentials in neural signaling of certain neural circuits.

## 6 Verification of the properties of neural information

To determine whether the above thesis that qualia and consciousness are forms of neural signaling information is correct, we must examine whether this type of information exhibits other properties outlined in Sections 3.5 to 3.8. If it does not, the current thesis will be wrong and must be rejected, and another entity that can underlie qualia and consciousness must be sought, potentially including explanations that require revisions or extensions of current physical theory.

### 6.1 Phenomenal property

Does information have the property of manifesting phenomenally as qualia or consciousness? Under the present framework, the answer is yes. As discussed in Section 2.3.4, theoretically, information can mean anything to a particular receiver in an appropriate circumstance. Hence, in theory, some neural information can mean a quale or consciousness of something to certain neural circuits under certain conditions. When those circuits read this information, they process and interpret it as such. Consequently, the information appears to them as that quale or consciousness, enabling them to incorporate something about it into their subsequent activities. This is how some information can become a quale or consciousness of something, or how a quale or consciousness of something occurs in certain neural circuits. Therefore, information can, in principle, have the property of manifesting phenomenally as qualia or consciousness, as required.

We can see more clearly how this happens in real life by examining how the visual information of a sunflower is transformed into a type that has this property. Consider Figure 1. The sunflower’s visual information enters the brain via the eyes. It is converted into neural information, i₁, and then processed through a sequence of neural circuits, resulting in progressively transformed information (i₂, i₃, i₄, …). Finally, a visual quale (Q) of the flower is generated by a certain group of neural circuits through some yet-unidentified neural processing mechanisms. As demonstrated in Section 5, this quale is most likely a form of information encoded in the patterns of action potentials in neural electrochemical signaling of some neural circuits; otherwise, it would not be processable, and awareness and experience of it—together with other neural activities about it, such as recognizing, remembering, and reporting its occurrence and phenomenal characteristics (e.g., its phenomenal shape, color, and brightness)—would not occur. But, in reality, these activities about the quale do occur. This means that several neural circuits process Q and interpret it as the flower visual quale. That is, Q does appear as the flower visual quale in the first-person view of these circuits, enabling them to generate activities about it. More concretely, this means that the patterns of neural signaling of Q have effects on neural circuits that read the patterns such that they appear in the processing systems of these neural circuits as the flower visual quale, enabling these circuits to produce outputs about the quale. This is how the physical visual information of the sunflower is transformed into a type of information that means the flower visual quale to some neural circuits and appears as such to them, giving rise to various activities about the quale. Effectively, this is how a quale occurs in the brain.

Although it may initially seem physically impossible or unlikely that neural information could appear phenomenally, it is essential to note that no novel entity emerges in the process. The appearance of the flower visual quale in some neural circuits is simply the appearance of the information that means this quale to them, when it is interpreted. It is not the creation of a new physical entity, nor does it involve changes of the physical nature of the entities in the process—input information, transformed information, and, finally, a quale—all are non-material entities with the same basic properties. If we examine our current physical laws, we find that none of them preclude information from being interpreted as something that manifests phenomenally—such as an image, sound, or smell—nor do they prohibit information from appearing as such to the circuits that interpret it correctly.

Furthermore, when we observe the information of a flower quale in neural circuits of other people from the outside, in the third-person perspective, we do not see the phenomenal flower quale. But if we could somehow become *the neural processing* of these circuits—reading and interpreting the information from the inside, from the first-person point of view—then a crucial question arises: What should we logically expect this information to appear to us as, especially given that we (as neural processing) incorporate something about the quale into our subsequent activities after its appearance?

Finally, a crucial question remains: How can neural circuits generate information that means phenomenal qualia or consciousness, and how can some neural circuits interpret such information correctly as phenomenal qualia or consciousness? To answer this question, it is crucial to note that this theory does not assume that all neural circuits possess such interpretive capacities. Rather, empirical evidence indicates that qualia and consciousness occur only in particular kinds of neural processing, predominantly in evolutionarily recent cortical systems, and not in more phylogenetically older structures such as the basal ganglia, brainstem, or cerebellum. This strongly suggests that certain neural circuits have acquired, through biological evolution, the functional capacity to generate information that carries phenomenal meaning and that certain other neural circuits have acquired the complementary capacity to interpret such information. When information with phenomenal meaning is processed by these specific circuits from the first-person perspective, it appears as qualia or consciousness. No new entities are introduced in this process; rather, phenomenality is the manner in which information with a particular meaning appears to the neural systems that are capable of interpreting it correctly. Crucially, this interpretation causes qualia or consciousness to appear privately only in the first-person view of neural processing—it does not cause qualia or consciousness to appear in the brain tissue or on its surface in the third-person view of external observers who observe that brain, which would be inexplicable under current physics.

Therefore, when examined more thoroughly, the idea that some information can appear phenomenally only in the first-person view of some neural circuits may no longer seem physically implausible.

### 6.2 Differential appearance property

Does information have the property of appearing differently from different points of view (PoVs)—manifesting phenomenally in one perspective but not in others? Under the present framework, the answer is affirmative. As discussed in Sections 2.3.3 and 2.3.4, if different information receivers receive the same information in dissimilar conditions, the effects, interpretations, and meanings generated in them are not the same. Thus, while a piece of information may mean something phenomenal, such as the flower visual quale, and appear as such to some neural circuits that directly read it, it may not have the same meaning or appear similarly in other receivers that indirectly receive it. To them, it may appear as something else, such as electrochemical activity.

This can be analyzed more comprehensively. Consider the flower visual quale, Q, in Figure 1. If Q is a form of information as proposed by this study, then for neural circuits that directly read this information, the information passes through synaptic junctions straight into their information-processing systems. There, it is processed and interpreted according to the meaning it carries for them—the flower visual quale. Consequently, it appears as such to them. In contrast, for external observers—such as investigators studying Q in someone who is looking at the flower—this information does not enter their information-processing systems directly. Instead, the information (in the form of electrochemical signals) first interacts with intermediary agents, such as EM waves, electric fields, magnetic fields, or sequences of these agents. Only afterward do the final mediated signals reach the observers’ information-processing systems and interact with them. Accordingly, external observers receive and process not the original information itself but its representations created by these mediators. This kind of indirect, mediated mode thus differs fundamentally from the direct, synaptic mode.

Because the information interacts with the first-person reading circuits and with the third-person external observers through different modes, the resulting effects, interpretations, and meanings differ (Sections 2.3.3 and 2.3.4). Thus, even though some information manifests phenomenally for the reading neural circuits, having the first-person PoV, the same information appears as something else, such as physical electrochemical activity, to external observers, having the third-person PoV. This explains why information can appear phenomenally to some neural circuits but not to external observers.

Finally, it should be noted that even if we or our most advanced instruments could somehow directly receive the information that carries the meaning of a quale or consciousness as first-person reading circuits do, the quale or consciousness would likely still not appear in our systems. Neither we nor our instruments possess the specialized decoding apparatus and processes required to correctly interpret that information as a quale or as consciousness. While some neural circuits evolved this capability, we and our instruments did not.

In conclusion, neural information possesses the property of differential appearance: It can manifest phenomenally in one perspective but not in others, as required.

### 6.3 Representational property

As discussed in Section 2.3.4, information can mean anything to receiving neural circuits in appropriate circumstances; hence, neural information can exhibit any representational content. For instance, it is obvious that, in the nervous system, information in the optic nerve can represent the visual aspect of an object; information in the auditory nerve can represent the auditory aspect of an air-vibration wave, and information in the olfactory nerve can represent the olfactory aspect of odorant molecules contacting the olfactory receptors. Thus, neural signaling information, when instantiated within modality-specific and functionally organized neural architectures, can exhibit representational properties, as required.

### 6.4 Other properties

#### 6.4.1 Intrinsic, private, and subjective

Information in a person always remains in that person because there is no neural link to share that information with other people. Although that person can form representations of that information, such as in the forms of verbal or written language, and share them with other people, the information itself cannot be shared. Therefore, information is intrinsic and private to that person, like qualia and consciousness, as required.

Additionally, since the meaning of information arises solely from how it is interpreted by the neural system of that person, the meaning of a piece of information and thus its appearance to that person (such as the shape, color, and brightness of the flower in that person’s mind) is inherently subjective, like qualia and consciousness, as required.

#### 6.4.2 Ineffable or indescribable

As discussed in Section 2.3.4, information can mean anything to receiving neural circuits in appropriate circumstances; hence, neural information can mean phenomena with different basic characteristics, such as images, sounds, smells, emotions, thoughts, and appears as such to the information receivers. Because their basic characteristics differ from others’, none of them can be described based on other phenomena. For example, one cannot describe what colors are like to congenitally blind people based on what sounds, smells, touches, or other phenomena are like; similarly, one cannot describe what sounds are like to congenitally deaf people based on what images, smells, touches, or other phenomena are like. Therefore, information can appear as phenomena that are indescribable or ineffable, as required.

#### 6.4.3 Complex, differentiated, and diverse in content and modality

Human neural information can be complex, differentiated, and diverse in content across modalities. This is because the organ that generates and processes information—the brain—is intricate and specialized enough. It is estimated to comprise approximately 86 billion neurons, with about 11.5 to 16 billion neurons in the cerebral cortex (Roth and Dicke, 2005; Herculano-Houzel, 2009). These neurons form extensive and complicated connections, resulting in 100 trillion (10^14^) and 1,000 trillion (10^15^) synapses (Flanagan, 1992, pp. 35–37; Roth and Dicke, 2005; Stiles and Jernigan, 2010). Neural signals encoded in various forms, such as rate, temporal, and mixed codes, are transmitted through these synapses (deCharms and Zador, 2000; Recce, 2001; Sanger, 2003; Rolls and Treves, 2011; Harris, 2013), resulting in various distinct states for each synapse. Theoretically, if each of these 100 trillion synapses can exist in at least 10 unique states, the total number of possible synaptic configurations in the brain would be on the order of 10^14^—i.e., 1 followed by 10^14^ or 100 trillion zeros. Remarkably, this number is incomparably larger than the estimated total number of elementary particles in the observable universe, which is approximately 10^87^, or 1 followed by 87 zeros (Flanagan, 1992, pp. 35–37). Therefore, even a small portion of the brain’s neural circuitry is capable of producing an unimaginable number of specific signaling states, each with different information, theoretically sufficient to represent virtually anything we can consciously experience—images, sounds, emotions, thoughts, memories, etc. (Flanagan, 1992; Tegmark, 2007).

Thus, the brain’s neural information can vary in complexity and content, and span multiple sensory modalities as required.

## 7 The hypothesis and predictions

We can see from the above analysis that neural signaling information possesses all the required properties listed in Section 3. In contrast, if we examine other entities in Groups I and II, we will find that they do not have some of these properties, especially the phenomenal and differential appearance properties, in addition to Property 3.4—being neurally processable—as discussed before. For example, presently, there is no evidence that neural electrical, magnetic, or EM signals can manifest phenomenally or appear differently across observers with different perspectives, despite affecting them similarly.

Because neural signaling information satisfies all the prerequisite properties of qualia and consciousness without requiring additional physical laws, whereas no other candidate entities have been found to do so, it is logical and parsimonious to conclude that every quale and every conscious state is a form of neural signaling information, and that each is the encoded signaling pattern of some specific group of neural circuits.

### 7.1 The hypothesis

This article formally submits the above conclusion as a hypothesis.

*Hypothesis: Every quale and every consciousness is a form of neural signaling information encoded in the signaling pattern of some specific group of neural circuits.*

Accordingly, based on the definitions in Sections 2.1 and 2.2, this hypothesis proposes that both the phenomena that manifest phenomenally in our minds (images, sounds, smells, emotions, thoughts, etc.) and our awareness and experiences of what these phenomena are like are forms of neural signaling information encoded in the signaling patterns of specific neural circuits. This type of information becomes phenomenal in some reading neural circuits through the process of information interpretation, as described in Section 6.1.

Importantly, to avoid misunderstanding, it should be noted that neural signaling information encoded in the signaling pattern of some neural circuits is not identical with the material electrical or chemical components of the signaling. Rather, it is the abstract, immaterial spatiotemporal pattern instantiated by those material components that constitutes the information.

### 7.2 Predictions

The above hypothesis provides empirically verifiable predictions. They are similar to those proposed in The Basic Theory of the Mind (Ukachoke, 2024, pp. 102–103, 133–134). They are also in line with the positions in other neuroscientific theories, such as Global Workspace Theory (Baars, 1988, 2005; Baars et al., 2013, 2021); Global Neuronal Workspace Hypothesis (Dehaene et al., 2003, 2011; Mashour et al., 2020), and Integrated Information Theory (Tononi, 2004, 2012; Oizumi et al., 2014; Tononi et al., 2016); however, the predictions here are stated explicitly and specifically. They are as follows:

1. A specific group of neural circuits, which are likely complex and distributed, will be found necessary and sufficient for the occurrence of a particular quale. This group is conceptually referred to as a single inclusive neural circuit termed a quale-generating neural circuit. A parallel prediction applies to consciousness, of which an associated group of neural circuits, probably even more complex and more distributed, is referred to here as a single inclusive neural circuit called the consciousness-generating neural circuit.

The qualia- or consciousness-generating neural circuit of a particular quale or consciousness can be identified by investigations that observe and may also manipulate potential groups of neural circuits concurrently with observing the quale or consciousness in question. In various investigations, the group that consistently changes concomitantly and correspondingly with the quale or consciousness will be the group that is the quale-generating or consciousness-generating neural circuit, respectively. This identification principle is the same as that employed by various studies of the NCC (see Introduction and Section 8.1).

1. A quale can be identified, quantified, or monitored by identifying, quantifying, or monitoring, respectively, only the signaling patterns of the quale-generating neural circuit. These actions on the signaling patterns are both necessary and sufficient for the corresponding quale’s investigations to result, while these actions on anything else without involving the signaling patterns will not result in the quale’s investigations. A parallel prediction holds for consciousness.
2. A quale can be created, modified, tested, or terminated by creating, modifying, testing, or terminating, respectively, only the signaling patterns of the quale-generating neural circuit. These actions on the signaling patterns are both necessary and sufficient for the corresponding actions on the quale to occur, and these actions on anything else without involving the signaling patterns will not result in the corresponding actions on the quale. A parallel prediction holds for consciousness.
3. The occurrence, change, and disappearance of the signaling pattern of a quale-generating neural circuit will be found to match exactly those of the quale. That is, in an event or experiment, all predictions that are valid for the signaling patterns of the quale-generating neural circuit, such as whether, when, or how they will occur, change, or disappear, will be identically valid for the quale; and vice versa. The same holds for consciousness and its associated circuit.

All the above predictions can be verified by experiments in conscious, communicative human subjects. A typical experiment is to monitor a quale or consciousness by having the subject report what happens to the quale or consciousness while concomitantly monitoring the signaling patterns of the qualia- or consciousness-generating neural circuit in the subjects by methods such as intracortical recording (see Mukamel and Fried, 2012; Homer et al., 2013; Wang et al., 2023), and while the signaling patterns of the neural circuit are being manipulated such as by drug administration, magnetic stimulation, or electrical stimulation.

Finally, it should be noted that the term “signaling patterns” in the above predictions refers to the spatiotemporal patterns of electrochemical signaling in the neural circuits being investigated. At present, the exact form of these “signaling patterns” is unknown. In the prior discussions (Sections 5.1 and 5.4), this study assumes that it is the patterns of all action potentials sent along all the axons because they are the strongest signals in neural electrochemical signaling (Bean and Koester, 2021; Siegelbaum and Fischbach, 2021), but it can be the patterns of presynaptic potentials, postsynaptic potentials, or other forms of presynaptic, synaptic, or postsynaptic activity. Because these patterns unfold one after another within the same neural architecture in rapid succession, they are likely to be closely correlated, meaning that determining one pattern may allow reasonable approximations of others, including the specific signaling patterns that underlie qualia and consciousness. Therefore, in principle, empirical research could proceed from coarse-grained approximations of any pattern toward more refined experimental designs progressively targeting the most likely patterns as technological capabilities advance. Nevertheless, although determining the exact form of the signaling patterns is certainly the ultimate goal in neuroscience, it is an empirical question and lies beyond the scope of the present article.

## 8 Discussion

Back to the analogy of a movie displayed on a television screen, as discussed before, the movie itself is not the screen. We can ascertain what it is from the fact that it consists only of the visual and audio information produced by the screen. Without this information, the movies would not exist—only when this information is generated, changed, or ceases being produced does this movie occur, change, or disappear, respectively. Therefore, it can be concluded that the movie physically is the visual and audio information—the patterns of EM waves and air vibrations—produced by the screen. Similarly, this study has shown that consciousness is not the neural correlates of consciousness (NCC) but a form of information—the patterns of some neural electrochemical signaling—generated by the NCC. Without this information, the consciousness would not exist, and only when this information is generated, changed, or ceases being produced does the consciousness occur, change, or disappear, respectively. In both cases, if we look at their circuits or physical signals from the sideline—or third-person perspective—we will not see the movie or the consciousness. Only when we read and interpret their information from the first-person perspective do the movie and the consciousness appear in our views. Remarkably, in both cases, no novel entity emerges in the processes to become the movie or the consciousness—they both simply are specialized forms of information with specific meanings, interpreted and appearing accordingly in their respective information-processing systems.

A possible objection to this kind of information-based accounts of consciousness is that the explanatory order may be reversed: Information may presuppose consciousness, rather than consciousness arising from information processing. On this view, information can be regarded as an abstract or observer-dependent construct introduced by consciousness-bearing agents. Some authors have argued in this line that physical activity does not by itself determine informational content, which instead depends on decoding frameworks or interpretive schemes (e.g., Haugeland, 1985; Worden, 2024). By contrast, other authors treat information as a real, functionally instantiated and causally efficacious feature of physical systems (e.g., Bateson, 1972; Landauer, 1999; Floridi, 2005, 2009; Dubrovsky, 2019; Krzanowski, 2020). Whether information should be understood primarily as an observer-dependent theoretical construct or as a real functional entity remains a matter of ongoing philosophical debate; however, a detailed examination of this issue lies beyond the scope of the present study.

Importantly, nevertheless, the result of this debate does not affect the empirical observation that consciousness does not occur in isolation, but only in association with particular forms of neural processing. The analysis presented here indicates that consciousness arises specifically from the processing of a non-material entity with content that is neurally processable, transferable, and causally effective, rather than from other forms of neural activity such as metabolic regulation, genetic expression, or structural maintenance. Whether this non-material entity is described as “information” or regarded as a theoretical construct does not alter what is empirically observed. Moreover, although humans in a conscious state can assign a name or concept to this non-material thing, consciousness is not the cause of it. Thus, although consciousness has a role in naming or conceptualizing this non-material thing, it is not its cause. The existence of this non-material does not require consciousness, either.

Finally, information interpretation in this theory is not an observer-relative semantic act, but a functional neural process. The outcome of this process is not pre-determined by the information (or neural signaling patterns), but is determined by the interaction among the information, the receiving neural circuits, and the contextual conditions of information transfer, as discussed in Sections 2.3.3 and 2.3.4. Only when these factors are appropriately aligned does the processing lead to the appearance of a quale or consciousness from the first-person perspective of the receiving neural circuits, enabling those circuits to incorporate the experienced content into subsequent neural activity. Thus, information interpretation in this framework arises from natural causal interactions among the information, the receiving neural circuits, and the context of processing. It is not a semantic interpretation based on an explicit symbol system, such as when a person encounters an unfamiliar written character (e.g., 意识) and consults a dictionary to interpret and assign it a meaning.

### 8.1 Theories and concepts on neural correlates of consciousness

As noted in the Introduction, numerous neuroscientific theories and frameworks address the neural correlates of consciousness (NCC). They generally propose that consciousness arises from large-scale neural activity involving global information availability, signal integration across distributed cortical and subcortical regions, and dynamic interactions between bottom-up and top-down processes. Mechanisms commonly invoked include reentrant processing, synchronous oscillations, gamma-band resonances, and predictive coding (Del Pin et al., 2021; Doerig et al., 2021; Sattin et al., 2021; Signorelli et al., 2021; Seth and Bayne, 2022; Storm et al., 2024; Cogitate Consortium et al., 2025). Representative theories include:

- Global Workspace Theory (GWT) (Baars, 1988, 2005; Baars et al., 2013, 2021).
- Neurobiological Theory of Consciousness (Crick and Koch, 1990).
- Theory of Neuronal Group Selection / Neural Darwinism (TNGS) (Edelman, 1993; Seth and Baars, 2005).
- The Neuronal Basis for Consciousness (Llinás et al., 1998).
- Recurrent Processing Theory (Lamme and Roelfsema, 2000; Lamme, 2006, 2010, 2018).
- Global Neuronal Workspace (GNW) (Dehaene et al., 2003, 2011; Mashour et al., 2020).
- Extended Global Workspace Theory (Song and Tang, 2008).
- Adaptive Resonance Theory (ART) (Grossberg, 2013, 2017).
- Predictive Processing Theory (Clark, 2013; Hohwy and Seth, 2020).

Other frameworks rest on fundamentally different premises. Orchestrated Objective Reduction (Orch OR) ties consciousness to quantum-level state collapses in microtubules (Hameroff and Penrose, 1996, 2014; Hameroff and Marcer, 1998; Hameroff, 2021). Operational Architectonics emphasizes the hierarchical, dynamical organization of neuronal assemblies (Fingelkurts and Fingelkurts, 2001; Fingelkurts et al., 2009, 2013). Conscious Electromagnetic Information (CEMI) and related electromagnetic field theories propose that consciousness corresponds to the brain’s electromagnetic information field (McFadden, 2002, 2013, 2020; Pockett, 2012). Integrated Information Theory (IIT) characterizes consciousness in terms of informational integration (Tononi, 2004, 2012; Oizumi et al., 2014; Tononi et al., 2016).

However, because identifying the physical nature of consciousness is not the primary objective of most of these theories, most of them typically do not specify explicitly what consciousness itself is physically. In many cases, this can only be inferred indirectly from how the theory describes the NCC.

By contrast, the present article focuses directly on identifying the physical nature of the entity to which the NCC correlates—consciousness—rather than further characterizing the NCC. Additionally, this article is purely theoretical and does not present new experimental data, whereas many NCC-focused theories do. Thus, this article’s hypothesis does not compete with existing theories of the NCC but offers a complementary clarification of the consciousness’s physical nature.

### 8.2 Theories and concepts on consciousness as a kind of neural pattern, aspect, or state

Regarding the physical nature of consciousness, several authors have proposed that consciousness is a particular kind of neural pattern, aspect, or state. Place (1956), for example, suggested that consciousness is a pattern of brain activity. Feigl (1958) similarly argued that conscious experiences are identical with certain configurational aspects of neural processes. Hameroff and Penrose (1996) introduced the Orchestrated Objective Reduction (Orch OR) theory, positing that consciousness arises from quantum state reductions within neuronal microtubules. Sevush (2006), in the Single-Neuron Theory of Consciousness, proposed that consciousness corresponds to spatial patterns of dendritic electrical activity in individual neurons. Graziano and Kastner (2011), Graziano and Webb (2015), and Graziano (2022), in the Attention Schema Theory, argued that consciousness is the brain’s internal model (or schema) of its own attentional processes. Grossberg (2013, 2017), within the Adaptive Resonance Theory (ART), held that all conscious states are resonance states. Loorits (2014) likewise suggested that consciousness is a certain complex pattern of neural activity.

However, these authors did not offer a solid rationale for how such neural patterns, aspects, or states come to manifest phenomenally as consciousness; phenomenal consciousness simply occurs. Also, they do not provide a basis for why the proposed patterns, aspects, or states manifest as phenomenal consciousness only in the first-person view but not in all views.

Therefore, although the physical nature of consciousness is addressed by these authors, their proposals are not the same as this article’s, which is that consciousness is a form of neural signaling information. Also, unlike the theories above, the present hypothesis provides explicit answers to the two key questions above (in Sections 6.1 and 6.2). These points distinguish the present hypothesis from prior theories that tackle this crucial matter.

### 8.3 Theories and concepts on consciousness as a form of information

Several authors have suggested that consciousness is a form of information, a view broadly aligned with the central claim of this article. For example, Dehaene and Naccache (2001) proposes that the global availability of information within a “global workspace” corresponds to the subjective experience of a conscious state. Tegmark (2007) suggests that consciousness is essentially “how information feels” when it is processed. Earl (2014) argues that consciousness consists purely of information in various forms. Dubrovsky (2019) proposes that the subjective phenomenon of something can be understood as information about that thing. Zhuravlev (2023) suggests that there seems to be a third level of information processing, which involves subjective consciousness and qualia. Other frameworks—including Chalmers’s Double-Aspect Theory of Information (Chalmers, 1995, 1997), Velmans’s Dual-Aspect Theory of Information (Velmans, 2002, 2009b), the Conscious Electromagnetic Information (CEMI) Field Theory (McFadden, 2002, 2013, 2020), the Information Closure Theory of Consciousness (ICT) (Chang et al., 2020), and The Basic Theory of the Mind (Ukachoke, 2024)—also treat consciousness as intimately related to information. However, most of these theories do not specify how information comes to manifest phenomenally, nor why it does so exclusively from a first-person perspective.

The Integrated Information Theory (IIT) (Tononi, 2004, 2012; Oizumi et al., 2014; Tononi et al., 2016) is one of the most influential information-based theories. It introduces the concept of integrated information (*Φ*) and proposes that a conscious experience corresponds to a maximally irreducible conceptual structure (MICS), which is specified by the subsystem that achieves the local maximum of integrated information (Φ_max). It uses these quantities to predict which systems are conscious, explaining, for instance, why consciousness is absent during sleep, generalized seizures, or in the cerebellum. Although computing Φ exactly is exceedingly complex, formal methods for estimating integrated information have been developed (Oizumi et al., 2014), and preliminary empirical estimates have been attempted in humans (Haun et al., 2017). Nevertheless, IIT does not provide a rationale for why MICS should give rise to phenomenal consciousness, nor why phenomenality occurs only in the first-person perspective (Mindt, 2017, 2025).

In contrast, this article does not provide a mathematical formula to quantify consciousness in any aspect and does not discuss the complexity of information in the generation of consciousness. Rather, it concentrates on the meanings and effects of information, as discussed in the preceding sections, particularly Sections 2.3.3, 2.3.4, and 6.1. Notably, according to this article’s hypothesis, the most crucial factor that determines whether any information is or can become phenomenal consciousness is not its complexity but its meaning, which corresponds to its effects. Additionally, this article clarifies why information can appear as phenomenal consciousness only in the first-person view of some neural circuits, as demonstrated in Section 6.2.

Therefore, this article refines existing information-based views by providing a neuroscientific rationale for how some information comes to manifest phenomenally as consciousness and why it does so only in the first-person perspective of certain neural circuits. It also proposes a new specific physical form of consciousness—information encoded in the spatiotemporal patterns of electrochemical signaling within certain neural circuits. Regarding The Basic Theory of the Mind (Ukachoke, 2024), which this study closely resembles, this study’s approach to the problem is more direct and methodical, and its explanation is more analytical and comprehensive.

### 8.4 Theories and concepts on consciousness’s differential appearances

The fact that qualia and consciousness do not appear to an external observer examining a person’s brain, but do appear within that person’s mind, raises the possibility that qualia and consciousness might be novel entities, with special characteristics, emerging inside that person’s brain. This matter has been addressed by many authors, for instance, Globus (1973), Velmans (1996, 2002, 2009b), Feinberg (2012), Feinberg and Mallatt (2016, 2020), and Solms and Friston (2018). These authors offer various parsimonious accounts suggesting that no novel entities emerge to constitute qualia or consciousness. Rather, the same qualia and consciousness simply appear differently from different points of view. The explanatory frameworks they propose nevertheless vary considerably, ranging from perspectival and access-based accounts to biologically grounded ontological views.

Velmans, for example, argues that a conscious experience and its correlated brain state are the same underlying event appearing differently depending on how it is accessed: First-person access reveals its phenomenal character, whereas third-person observation reveals its neural properties. These perspectives are complementary and mutually irreducible (Velmans, 1996, 2002, 2009b). Solms and Friston (2018) locate the distinction within the intrinsic organization of self-organizing biological systems. On their active-inference view, first-person experience reflects the system’s internal, affective monitoring of its own states, whereas third-person descriptions portray the same dynamics in neural, computational, and thermodynamic terms. Feinberg and Mallatt argue that subjective experience emerges from evolutionarily ancient neural architectures that integrate multisensory representations with affective valuation. For them, first-person properties such as sensory qualia and feeling are real, irreducible features of these representational systems, while third-person accounts describe the neurobiological mechanisms that make such states possible (Feinberg, 2012; Feinberg and Mallatt, 2016, 2020). Although differing in emphasis, these approaches converge on the view that subjective experience and its neural correlates are two inseparable sides of the same process, and that neither perspective alone is sufficient for a complete explanation.

However, although these frameworks provide valuable conceptual insight into why first-person and third-person descriptions differ without positing any novel emergent entities, one important question remains unanswered: What is the neural mechanism by which something appears phenomenally from the first-person perspective?

This article offers a clarification as discussed in Sections 6.1 and 6.2. Shortly, consciousness is neural information that means “consciousness” to some neural circuits. When this information is read from the first-person PoV by those circuits, it is interpreted accordingly; hence, consciousness appears in that view. By contrast, when observed from the third-person PoV by external observers, it is not properly interpreted like in the first-person case; thus, consciousness does not appear in this view. Therefore, this article’s concept aligns with the general insight of other authors that consciousness has differential appearances from different perspectives, but it also specifies the neural mechanism by which information manifests phenomenally only when read by the appropriate first-person circuits.

### 8.5 Implications and limitations

#### 8.5.1 Practical applications

If consciousness is a form of neural information instantiated by specific neural signaling patterns, then in principle it should be possible to identify the content of a conscious state objectively by comparing the signaling patterns associated with it to patterns corresponding to known conscious experiences. This could allow objective determination of whether a conscious state contains an image, sound, taste, emotion, or thought—and potentially which specific one (e.g., an image of a flower, a person’s face, or a building)—by referencing a catalog of neural signaling patterns for known conscious contents.

Furthermore, tracking the emergence, transformation, and disappearance of these signaling patterns could enable objective monitoring of consciousness in both research and clinical settings. Such capabilities would assist in evaluating consciousness in non-communicative patients. They could also help determine when consciousness first arises in fetuses. Searching for comparable signaling patterns in non-human animals can assist in resolving whether non-human animals possess some forms of consciousness analogous to human consciousness, and, if some do, which ones do.

In addition, the physical parameters of neural signaling patterns—such as the number of neurons involved, the strength of associated electrical and magnetic fields, and the characteristics of any accompanying electromagnetic emissions—could theoretically provide quantitative measurements of conscious states. These might form the basis for “consciousness metering” devices capable of objectively assessing the intensity of particular experiences, such as pain, pleasure, or emotional states.

#### 8.5.2 Theoretical applications

If the physical nature of consciousness is known, then how it interacts with other physical entities can become clearer. If consciousness is a form of information, as proposed here, and given that the physical and mathematical properties of information are comparatively well understood, several long-standing theoretical questions may be addressed more effectively from a neuroscientific standpoint. These include whether consciousness is epiphenomenal and why conscious experience occurs in the brain at all—questions commonly known as the “epiphenomenalism problem” (Chalmers, 1996; Weisberg, 2012; Robinson, 2023) and the “hard problem of consciousness” (Chalmers, 1995; Libet, 1996; Velmans, 2009b; Weisberg, 2012; Van Gulick, 2017), respectively.

Another important implication concerns the potential for consciousness in non-biological systems. If consciousness is simply a form of information that means consciousness to certain receivers, then consciousness could theoretically arise in any physical system in which some information carries such meaning to some of its parts. When these parts interpret that information as consciousness and incorporate aspects of it into their downstream activity, consciousness arises in that system. Thus, this article’s framework, like The Basic Theory of the Mind (Ukachoke, 2024, pp. 166–170), provides a general physical mechanism for consciousness in any system.

Consequently, if we learn how to generate information that means consciousness to some of their components in silicon-based processors, it can become possible to create artificial consciousness in robots and other forms of silicon-based AI. Detailed knowledge of the neural signaling patterns underlying human consciousness and of the structure and processes of the consciousness-generating neural circuits could provide the foundation for such work.

#### 8.5.3 Limitations

This article has several limitations. The most notable ones are:

1. This study is purely theoretical and based entirely on existing evidence; it does not present additional empirical evidence supporting its framework.
2. Although this study proposes the existence of a consciousness-generating neural circuit and of the specific neural signaling pattern underlying consciousness, it does not identify that neural circuit or the exact form of that signaling pattern.
3. Although this article proposes that, in the process of creating consciousness, a certain group of neural circuits generates information that means “consciousness” to certain receivers and that those receivers can subsequently interpret that information correctly as “consciousness,” the neural processes underlying such information generation and subsequent interpretation are not identified by this study.
4. The hypothesis’s predictions are likely challenging to verify empirically with high precision, given current technological limitations.
5. This study’s analysis depends on the working definitions of qualia, consciousness, and information adopted in Section 2. Although these definitions are grounded in existing literature and refer to common phenomena we experience in our daily life, they differ from some other formulations. Accordingly, the hypothesis and its implications apply only within the conceptual framework established on these working definitions.

Despite these limitations, the hypothesis provides a new, coherent, and parsimonious neuroscientific framework for understanding the physical nature of consciousness. Therefore, these limitations serve not as its drawbacks but as guides for future empirical and theoretical investigation and refinement.

## 9 Conclusion

This study presents a neuroscientific hypothesis on the physical nature of consciousness. It develops the hypothesis by examining the essential properties of consciousness, identifying potential entities that could underlie consciousness, and analyzing how neural circuits function in generating consciousness. Each potential entity is then evaluated for whether it is processable by neural circuits and whether it possesses all the required properties of consciousness. Based on these investigations and the principle of parsimony, the study concludes that neural information underlies consciousness and that consciousness is physically a form of neural information—specifically, information encoded in the spatiotemporal patterns of electrochemical signaling within certain neural circuits. The study also provides neuroscientific explanations for how such information can manifest phenomenally as consciousness and why this manifestation occurs only from the first-person perspective of some neural circuits. The hypothesis yields empirically testable predictions, making it falsifiable. Since it does not posit any novel entity, force, or physical law and since all evidence and arguments remain within the boundaries of established neuroscience, the hypothesis is parsimonious and neuroscientific.

In relation to existing theories of consciousness, this hypothesis aligns with many of them on several important points, as discussed in the previous section. Its principal contributions are: (1) it proposes a new specific physical form for consciousness, (2) it presents a neuroscientific explanation of how consciousness arises in the brain, and (3) it identifies a neural mechanism for why consciousness appears only from the first-person perspective.

Although the hypothesis has several limitations, as previously discussed, it is hoped that the framework, analyses, and explanatory models developed here can provide useful concepts for further empirical and theoretical research on the nature of consciousness.

## Data Availability

The original contributions presented in the study are included in the article/supplementary material, further inquiries can be directed to the corresponding author.

## Footnotes

1 In this article, “*non-material*” means “*not composed of elementary particles with mass (e.g., fermions)*,” and “*material*” means “*composed of such particles*.” Importantly, “*non-material*” does not imply non-physical or supernatural status. Such entities remain fully within the domain of physical law. For example, electromagnetic waves, electrical fields, and magnetic fields are non-material in this technical sense, yet they are entirely physical and governed by established physical principles.

2 In this article, “*content*” means “*something non-material that is about some object, event, issue, or other things*.”

3 In this article, when “*read*” is used with neurons, neural circuits, or other agents interacting with a piece of information, it means “*to take the information into their information-processing systems*.”
